# Birth Weight and Labor Market Outcomes: Findings From Tohoku Medical Megabank Data

**DOI:** 10.1002/puh2.221

**Published:** 2024-08-19

**Authors:** Midori Matsushima, Taku Obara, Mami Ishikuro, Naoki Nakaya, Atsushi Hozawa, Shinichi Kuriyama

**Affiliations:** ^1^ Faculty of Humanities and Social Sciences University of Tsukuba Tsukuba Japan; ^2^ Graduate School of Medicine Tohoku University Sendai Japan

**Keywords:** birth weight, disaster, labor market outcomes, recovery phase

## Abstract

**Background:**

Epidemiological and economic literature has revealed that low birth weight (LBW) is related to poor health conditions and a broader range of negative socio‐economic outcomes, including academic achievement, income levels, and working status. However, Japanese evidence for labor outcomes is particularly lacking, as is the question of whether the impact of LBW on adulthood worsens when disasters occur. We aimed to reveal the impact on LBW on labor outcomes during the disaster recovery phase.

**Methods:**

We used a cross‐sectional data set of 4156 national health insurance holders, males aged 40–60 years, from the Miyagi and Iwate prefectures during the earthquake recovery phase. Employing a multinomial probit and logistic model, we estimated the impact of LBW on the employment status, changes in income, and the propensity to face a decline in income with a job change.

**Results:**

The very low birth weights (VLBWs) have a disadvantage in all labor market outcomes. Compared to the non‐LBWs, the VLBWs were 15.2% less likely to be full‐time/self‐employed and 17.1% more likely to be contingent/temporary/part‐time workers. Moreover, 32.9% are more likely to face a decline in income and approximately 3.7 times more likely to change jobs leading to income decline. The LBWs were likely to face a decline in income by 8.6% and 4.6%, respectively, but no other significant effects were found on other outcomes.

**Conclusions:**

The effects were apparent for the VLBW, but not so much for the LBW. The effects became larger during the disaster recovery phase, even though the regional economies were boosted.

## Graphical abstract

We aimed to reveal the impact on LBW on labor outcomes during the disaster recovery phase. Two findings should be highlighted. First, the effects were apparent for the VLBW, but not so much for the LBW. Second, the effects became larger during the disaster recovery phase.

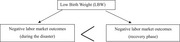

**Abbreviations**
COVID‐19: coronavirus disease
LBW: low birth weight
VLBW: very low birth weight

## 1 Introduction

Epidemiological research has revealed that poor health at the time of birth can trigger chronic, degenerative health conditions during adulthood. Economists have further explored the impact of newborn health, generally measured by birth weight, on a broader range of outcomes, including academic achievement, income levels, and working status; per their findings, poor health of newborns is linked with negative consequences in the western society [1, 2, 3, 4]. This is explained by the fetal origins hypothesis, also known as Barker's hypothesis, and later the developmental origins of health and disease hypothesis [5]. The hypothesis states that when a fetus is exposed to an adverse environment in utero, the baby is likely to be born unhealthy (often measured by birth weight), which negatively impacts the entire lifespan due to scheduled prenatal brain and body development deficiencies [6, 7, 8, 9]. Grossman's model suggests that one's health determines the total amount of time one can spend acquiring monetary earnings and commodities [10]; thus, if poor health begins from birth, one is likely to underperform in terms of socio‐economic outcomes. Additionally, educational achievement is vital in most societies for acquiring a job. Thus, having low educational qualifications leads to poor labor outcomes.

In Japan, evidence is limited due to the lack of longitudinal data. However, taking results of all previous research into account, we find that, in general, the effects exist, particularly for health outcomes, and the magnitude of impact on human capital, academic achievement, and labor outcomes is negligible [11, 12]. This study aimed to increase knowledge of the effects of birth weight on labor market outcomes by focusing on middle‐aged men in the aftermath of the Great East Japan Earthquake (2013–2016).

This study is unique as we used data of male national health insurance holders who were living in the disaster‐stricken area, and we estimated the impact of birth weight on employment status, job, and income stability. This implies that we are conducting our study in a sample that is in the recovery phase postcrisis. The earthquake resulted in large‐scale destruction of the local economy and a shortage of healthcare services. However, by 2013, the disaster‐stricken area had started to recover, and the demand for reconstruction boosted the labor demand, resulting in an increase in the average wages. Additionally, the indicator of the Private Consumption Integrated Estimates recovered. Indeed, due to large public subsidies for recovery, regional domestic expenditure postdisaster more than doubled compared to the regional domestic expenditure predisaster; the disaster‐stricken area also showed the best performance in the past 10 years with respect to three out of four indicators, namely, the overall regional expenditure indicator, the regional private companies’ infrastructure investment indicator, and the regional public investment indicator [13]. During the first half of 2015, the reconstruction demand started to slow down with a decline in public work related to infrastructure reconstruction. Nevertheless, many regional economic indicators still showed higher economic performance compared to pre‐earthquake times, as a number of effective job offers remained high [14].

However, neither the effects of crises nor the fruits of recovery are equally available to everyone. Although we are experiencing different types of crises, there is a commonality between the two: economic impact and limited access to healthcare services. Learning from the experience of the Great East Japan Earthquake, we can obtain implications for our current society.

## 2 Methods

### 2.1 Study Design and Study Population

**FIGURE 1:**
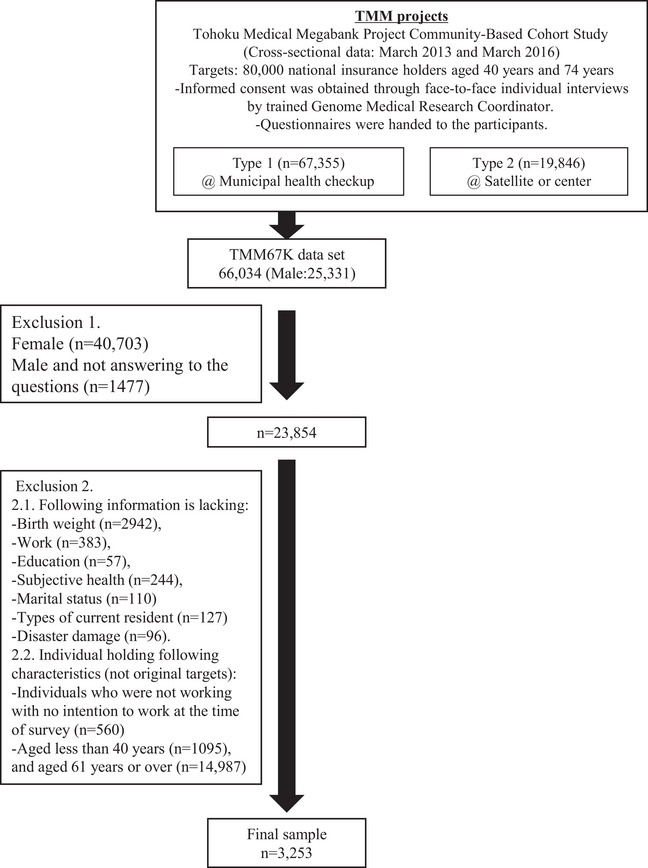
Flow chart of our sampled data.

We used the cross‐sectional data set from Type 1 survey in the flow chart (Figure 1), which were conducted between March 2013 and March 2016, targeting national health insurance holders who came to the municipal health checkup. Inclusion criteria were defined as persons aged 20 years and over and 75 years and under, registered in the basic resident register of all municipalities in Miyagi Prefecture and 20 municipalities in Iwate Prefecture at the time of enrollment for the specific health checkup sites. The exclusion criteria were (1) persons who do not consent to participate in the study and (2) persons who are not able to fill out study questionnaires [15]. For further detail, see Hozawa et al. [16]. By the end of March 2016, 97,419 individuals had been recruited in Type 1 survey, and 67,355 persons had participated (a response rate of 69%). From this data set, the Tohoku Medical Megabank Organization (ToMMo) provided TMM67K data set, which includes 66,034 individuals (1321 individuals were excluded due to missing basic demographic information and general health information). Among them, female respondents (*n* = 40,703) were excluded, as were 1477 male respondents who did not return some parts of the questionnaires.

### 2.2 Data Sources

The data set we used was from the Tohoku Medical Megabank Project Community‐Based Cohort Study, which was a part of The Tohoku Medical Megabank Project (TMM). The description of the whole project is summarized in Kuriyama et al. [15], and its activities are periodically updated on the webpage of the Tohoku University, Tohoku Medical Megabank Organization (ToMMo) (ToMMo, n.d) (https://www.megabank.tohoku.ac.jp/activity). The pilot study was not conducted for the validation of the questionnaire, but the survey questionnaire used in this survey was created based on validated questionnaires, and it was designed to be comparable to Japanese previous studies such as JPHC (the Japan Public Health Center‐based Cohort) or JMICC (the Japan Multi‐institutional Collaborative Cohort).

We used data of 4156 national health insurers’ males aged 40–60 years from the Miyagi and Iwate prefectures between 2013 and 2016. Our variable of interest is the birth weight: less than 1500 g, 1500–1999 g, 2000–2499 g, and the others. We first employed a multinomial probit model to estimate the impact of birth weight on the employment status. Next, we also used a multinomial probit model to understand the effect of birth weight on changes in income after the earthquake. Lastly, we used logistic models to examine the propensity to change the job and decline in income with a change in job during the aftermath of the earthquake.

A critical point here is that the data used in this study are mainly the data of the national health insurance holders. The national health insurance is designed to cover the self‐employed, the unemployed, and people who are not covered by any other health insurance, including seamen's insurance or mutual aid association plans. Moreover, part‐time/seasonal workers without a certain period of employment in applicable business establishments are included in this insurance. This characteristic of our data indicates that although our results may not be applicable to the employed people, they have the advantage of understanding the impact of poor health at birth on labor outcomes of the self‐employed in the aftermath of the earthquake, which is unique because the proportion of the self‐employed people among the general population survey data is small and focusing on only this subgroup is not suitable for regression analysis.

In order to analyze data, we cleaned our data set as follows. A total of 23,854 respondents, we excluded the individuals who were unable to provide information on the measurements we needed, that is, birth weight (*n* = 2942), work (*n* = 383), education (*n* = 57), subjective health (*n* = 244), marital status (*n* = 110), types of current residents (*n* = 127), and disaster damage (*n* = 96). Additionally, we excluded individuals who were not working with no intention to work at the time of the survey (*n* = 560), and the ones aged less than 40 years (*n* = 1095), and those aged 61 years or over (*n* = 14,987). The final sample included 3253 participants.

### 2.3 Measures

#### 2.3.1 Labor Market Outcome Variables

We used four outcome variables: *working status*, *income change*, *job changes*, and *job change with income decline*. *Working status* is a variable with three possible statuses: self‐employed/full‐time workers, contingent/temporary/part‐time workers, and unemployed. *Income change* also has three outcomes: increased income, decreased income, and no changes in income. *Job change* is a binary variable of: 1—changed the job after the earthquake; 0—did not change the job. *Job change with income decline* is also a binary variable of: 1—changed the job after the earthquake along with a decline in income; 0—otherwise.

#### 2.3.2 Birth Weight Variable

Our focused variable is birth weight. Birth weight was recorded by means of a question given to the respondents. The response options were categorical—1: <1500 g, 2: 1500 < 2000 g, 3: 2000 < 2500 g, 4: 2500 < 3000 g, 5: 3000 < 3500 g, 6: 3500 < 4000 g, 7: 4000 g**≥**, and 8: Do not know. In our analysis, we created the dummy variables “<1500g,” “1500 < 2000 g,” “2000 < 2500 g,” and “2500 g**≥**.” We named them *birth weight 1500*, *birth weight 2000*, *birth weight 2500*, and *birth weight over 2500*, respectively. Birth weight less than 1500 g is generally considered very low birth weight (VLBW), and 2500 or less is considered low birth weight (LBW). In Japan, in addition to this medical categorization, there is another threshold of 2000 g, which is defined by medical care benefits for premature babies. In Japan, when babies are born with a birth weight of 2000 g or less, they are entitled to receive the medical care benefits for premature babies, which cover medical costs for infants up to 1 year of age. Hence, we created variables with the thresholds of 1500, 2000, and 2500 g.

#### 2.3.3 Covariates

Covariates include educational attainment, marital status, number of children, age of the respondent, self‐rated health, disaster damage, and the types of current residents. As for educational attainment, we defined the variable as 1: undergraduate or higher level of education, and 0: lower than an undergraduate education. Marital status was defined as 1: currently having a partner, and 0: not having a partner. Number of children and age of the respondent were included in the regression model as continuous variables. Self‐rated health is included to control health effects on employment, and the variable is the ordered categorical variable—1: not good at all, 2: not so good, 3: good, 4: very good. Disaster damage was used as a dummy variable, and each variable captured the condition of respondents’ residence: completely destroyed, mostly destroyed, partially destroyed, partially damaged, no damage, and not living in the disaster‐stricken area. Types of current residents were also included as dummy variables: temporary housing built by the government, temporary housing subsidized by the government, a rented house/apartment, a house/apartment owned by family, friends, and acquaintances, a house built in the same dwelling as before (the house was destroyed due to the earthquake), a house built in a different dwelling from before (the house was destroyed due to the earthquake), a house the same as before the earthquake (the house was not destroyed/completely destroyed), and others. In addition to these covariates, the survey year was controlled in the analysis.

#### 2.3.4 Statistical Analysis

In order to estimate the effect of birth weight on working status, we first consider the choices given to the individuals: (1) self‐employed/full‐time workers, (2) contingent/temporary/part‐time workers, and (3) the unemployed. Because the independence of irrelevant alternatives assumption was not being satisfied, we used a multinomial probit model. Thus, the estimation model is written as

(1) $$\begin{array}{ccc} & & working\_status_{di}=\beta_{1}birthweight\;1500_{di}\\ & & \;+\;\beta_{2}birthweight\;2000_{di}+\beta_{3}birthweight\;2500_{di}\\ & & \;+\;\beta_{4}X_{di}+\varepsilon_{di} \end{array}$$

where $working\_status_{di}$ is the labor outcome of the individual who chose one of the above three mentioned choices *“d*.” Birth weight was included as each dummy variable with the reference to birth weight of 2500 g or over, and $\beta_{1}$, $\beta_{2}$, $\beta_{3}$, and $\beta_{4}$ represents marginal effects of each variable to choose the labor outcomes, respectively. $X_{di}$ is the set of covariates mentioned earlier.

Second, we also used a multinomial probit model to explore the effects of birth weight on income after the earthquake. Variables of birth weight and covariates are the same as in Model (1) with a different outcome variable of *Income change*. It also has three outcomes: (1) increased income, (2) decreased income, and (3) no changes in income:

(2) $$\begin{array}{ccc} & & Income_{c}hange_{di}=\beta_{1}birthweight\;1500_{di}\\ & & \;+\;\beta_{2}birthweight\;2000_{di}+\beta_{3}birthweight\;2500_{di}\\ & & \;+\;\beta_{4}X_{di}+\varepsilon_{di} \end{array}$$

Finally, we explored the propensity of each birth weight category to face unfavorable labor and economic conditions in the aftermath of the earthquake with two binary variables of *job changes* and *job changes with income decline*:

(3) $$\begin{array}{ccc} Job\_changes_{i} & = & \beta_{1}\;birthweight\;1500_{i}+\beta_{2}birthweight\;2000_{i}\\ & & +\;\beta_{3}birthweight\;2500_{i}+\beta_{5}X_{i}+\varepsilon_{i} \end{array}$$

(4) $$\begin{array}{ccc} & & \mathrm{Job}\_\mathrm{changes}\_\mathrm{income}\_\mathrm{declin}e_{i}=\beta_{1}\;birthweight1500_{i}\\ & & \;+\;\beta_{2}birthweight\;2000_{i}+\beta_{3}birthweight2500_{i}\\ & & \;+\;\beta_{5}X_{i}+\varepsilon_{i} \end{array}$$

In Models (3) and (4), the outcome variables were binary and captured details on whether the respondent had to change his job due to the earthquake, and whether he experienced job changes accompanied with income decline after the earthquake. In both models, odds ratios of $birthweight1500_{i},\;birthweight\;2000_{i},=$ and $\;birthweight2500_{i}$ are shown with the reference to birth weight 2500 g or over.

STATA MP‐17, Texas, was used for analyses.

## 3 Results

The key features of the sample of this study are summarized in Table 1. There are several notable points. Educational attainment was lower than the Japanese average, as Census 2010 shows about 29% of males had at least an undergraduate level education or more. This may be attributable to the uniqueness of our data collection, and we need to take these characteristics into account when discussing the results. With regard to earthquake‐related information, the majority of the respondents faced some disaster damage. At the time of the survey, they stayed in the same place as before the earthquake, and about 8% still lived in temporary housing.

**TABLE 1:** Key features of the study sample.

	All	<1500g	1500 < 2000 g	2000 < 2500 g	2500 g or over
	*n* = 3253	*n* = 20	*n* = 110	*n* = 349	*n* = 2774
Education (less than undergraduates)	2757 (84.8%)	18 (90.0%)	96 (87.3%)	308 (88.3%)	2335 (84.2%)
Education (undergraduates or over)	496 (15.2%)	2 (10.0%)	14 (12.7%)	41 (11.7%)	439 (15.8%)
Not having a partner	988 (30.4%)	7 (35.0%)	38 (34.5%)	103 (29.5%)	840 (30.3%)
Having a partner	2265 (69.6%)	13 (65.0%)	72 (65.5%)	246 (70.5%)	1934 (69.7%)
Number of children	0.1 (0.4)	0.0 (0.0)	0.2 (0.6)	0.1 (0.4)	0.1 (0.4)
Age of the respondent	50.9 (6.1)	51.0 (7.3)	51.9 (5.9)	51.8 (6.1)	50.8 (6.1)
Self‐rated health	2.9 (0.5)	2.8 (0.6)	2.9 (0.6)	2.9 (0.6)	2.9 (0.5)
***Disaster damage***					
Completely destroyed	354 (11.2%)	1 (5.3%)	14 (12.8%)	33 (9.9%)	306 (11.4%)
Mostly destroyed	113 (3.6%)	2 (10.5%)	6 (5.5%)	6 (1.8%)	99 (3.7%)
Partially destroyed	231 (7.3%)	0 (0.0%)	6 (5.5%)	20 (6.0%)	205 (7.6%)
Partially damaged	1151 (36.4%)	13 (68.4%)	37 (33.9%)	139 (41.5%)	962 (35.7%)
No damage	1119 (35.4%)	3 (15.8%)	42 (38.5%)	113 (33.7%)	961 (35.6%)
Not living in the disaster area	191 (6.0%)	0 (0.0%)	4 (3.7%)	24 (7.2%)	163 (6.0%)
***Types of the current residence***					
A temporary housing built by the government	174 (5.5%)	1 (5.0%)	9 (8.4%)	17 (5.1%)	147 (5.4%)
A temporary housing subsidized by the government	64 (2.0%)	0 (0.0%)	3 (2.8%)	7 (2.1%)	54 (2.0%)
A rented house/apartment	50 (1.6%)	0 (0.0%)	3 (2.8%)	4 (1.2%)	43 (1.6%)
A house/apartment owned by family, friends, and acquaintances	81 (2.5%)	1 (5.0%)	3 (2.8%)	10 (3.0%)	67 (2.5%)
A house built in the same dwelling as before	111 (3.5%)	1 (5.0%)	1 (0.9%)	9 (2.7%)	100 (3.7%)
A house built in a different dwelling from before	94 (3.0%)	0 (0.0%)	3 (2.8%)	6 (1.8%)	85 (3.1%)
A house same as before the earthquake	2483 (77.9%)	16 (80.0%)	83 (77.6%)	274 (81.5%)	2110 (77.5%)
Others	129 (4.0%)	1 (5.0%)	2 (1.9%)	9 (2.7%)	117 (4.3%)

Table 2 shows a comparison of the prevalence of labor outcomes between birth weight status. With regard to labor outcome variables, we can see the differences in full‐time/self‐employed, as a less percentage of people who belong to full‐time/self‐employed category are among those with lower birth weights. Moreover, the prevalence of contingent/temporary/part‐time between groups differs, as a higher percentage of people who belong to that category are among those with lower birth weights. Although overall, the income change variable showed that the majority of the respondents experienced no change in income (43.7%), followed by a group that experienced a decrease (38.0%), and a small minority of respondents experienced an increase in income levels postdisaster (18.3%), lower birth weighted ones have faced income decline. As the indicator of job change suggest, most people who changed the job were experiencing income decline.

**TABLE 2:** Prevalence (%) of labor outcomes by birth weight status.

	<1500 g	1500 < 2000 g	2000 < 2500 g	2500 g or over	*p*‐value
***Working status***	*n* = 20	*n* = 110	*n* = 349	*n* = 2774	
Full‐time/Self‐employed	12 (60.0%)	85 (77.3%)	294 (84.2%)	2293 (82.7%)	0.02
Contingent/Temporary/Part‐time	7 (35.0%)	18 (16.4%)	36 (10.3%)	302 (10.9%)	0.002
Unemployed	1 (5.0%)	7 (6.4%)	19 (5.4%)	179 (6.5%)	0.90
***Labor outcomes***	*n* = 19	*n* = 103	*n* = 330	*n* = 2595	
Income increased	2 (10.5%)	17 (16.7%)	61 (18.7%)	483 (18.8%)	0.78
Income decreased	13 (68.4%)	48 (47.1%)	130 (39.8%)	969 (37.6%)	0.010
Income no change	4 (21.1%)	37 (36.3%)	136 (41.6%)	1124 (43.6%)	0.098
***Changes in job and income decline***	*n* = 19	*n* = 103	*n* = 330	*n* = 2595	
Job changes	7 (36.8%)	23 (22.3%)	60 (18.2%)	474 (18.3%)	0.15
Job changes with income decline	6 (31.6%)	16 (15.5%)	35 (10.6%)	310 (11.9%)	0.034

### 3.1 Work Status

The marginal effects of birth weight on working status are shown (Table 3). The VLBWs were significantly more likely to have a disadvantage in labor market outcomes compared to the ones who weighed 2500 g or more at birth. They were 15% less likely to be full‐time/self‐employed, and 17% more likely to be contingent/temporary/part‐time workers. The working status of the respondents who weighed 1500 g or more at birth was indifferent from respondents with non‐LBWs.

**TABLE 3:** Birth weight and working status (Marginal effects).

	Full‐time/Self‐employed			Contingent/Temporary/Part‐time			Unemployment	
	***dy*/*dx***		SE	***dy*/*dx***		SE	***dy*/*dx***	SE
<1500 g	−0.15	^**^	(0.07)	0.17	^***^	(0.06)	−0.02	(0.06)
1500 < 2000 g	−0.03		(0.04)	0.04		(0.03)	−0.01	(0.02)
2000 < 2500 g	0.01		(0.02)	0.00		(0.02)	−0.01	(0.01)

*Note:* All the other factors are held constant. Standard errors in parentheses.

* *p* < 0.10.

** *p* < 0.05.

*** *p* < 0.01.

### 3.2 Income Decline

Table 4 shows the marginal effects of birth weight on income decline after the earthquake. Compared to respondents who weighed 2500 g or more at birth, respondents born with a weight of less than 2000 g had a significantly higher likelihood of facing income decline after the earthquake. The marginal effects were largest for respondents with a birth weight of born less than 1500 g (0.32), followed by 1500 < 2000 g (0.09). The statistical significances were 5% and 10%, respectively. Although insignificant, the marginal effect of 2000 < 2500 g is 0.04. As for no change in income, born less than 1500 g is less likely to be in that category by 26%. Considering the signs of other marginal effects, the results support the robustness that respondents with LBW had a higher probability of income decline after the disaster.

**TABLE 4:** Birth weight and income decline.

	Income increased		Income decreased		No change	
	*dy*/*dx*	SE	*dy*/*dx*	SE	*dy*/*dx*	SE
<1500 g	−0.06	(0.11)	0.32^***^	(0.12)	−0.26^*^	(0.14)
1500 < 2000 g	−0.03	(0.04)	0.09^*^	(0.10)	−0.53	(0.30)
2000 < 2500 g	−0.00	(0.02)	0.04	(0.20)	−0.04	(0.03)

*Note:* All the other factors are held constant. Standard errors in parentheses.

* *p* < 0.10.

** *p* < 0.05.

*** *p* < 0.01.

### 3.3 Job Changes and Income Decline

Table 5 shows the results of logistic regressions and the odds ratios of each variable. Compared to respondents who weighed 2500 g or more at birth, those who weighed less than 1500 g at birth had a 2.8 times higher likelihood of changing their jobs. The odds ratio became higher when job change was accompanied with income decline (3.71).

**TABLE 5:** Birth weight and job change, income decline.

	Changed job after the earthquake		Changed job after the earthquake and income decreased	
	Odds ratio	SE	Odds ratio	SE
<1500 g	2.80^**^	(1.43)	3.71^**^	(1.93)
1500 < 2000 g	1.26	(0.32)	1.33	(0.38)
2000 < 2500 g	1.10	(0.18)	0.94	(0.19)

*Note:* All the other factors are held constant. Standard errors in parentheses.

* *p* < 0.10.

** *p* < 0.05.

*** *p* < 0.01.

## 4 Discussion

Previous studies in Japan did not find evidence of LBW being linked with poorer educational or labor outcomes in adulthood [11, 12]. Our study has investigated whether this trend holds during the reconstruction phase. Per the results of our analyses, VLBW has significant adverse impacts on labor market outcomes during middle‐age. These respondents had a lower likelihood of being self‐employed/full‐time workers and a higher likelihood of being contingent/temporary/part‐time workers. Additionally, they were likely to have experienced income decline and job changes accompanied by income decline after the earthquake. Although we were unable to identify whether the respondents with VLBW were not being full‐time/self‐employed only after the earthquake or if it had the same trend before the earthquake, we can claim that respondents with VLBWs were particularly vulnerable. On the other hand, respondents who weighed at least 1500 g at birth had to face income decline, yet the effect on other variables was insignificant. These results suggest that the magnitude of impact differs even among the respondents with LBW, and during the aftermath of crises, respondents with VLBW can face greater difficulties. It must be noted that the timing of the survey was during the recovery phase of the disaster area. The regional economy was boosted, and it performed even better compared to the prepandemic period. This indicates that those who were vulnerable were likely to be left behind from the recovery.

There are several paths we can think of that affect labor outcomes. The meta‐analysis by Aarnoudse‐Moens et al. suggests that babies with VLBW were likely to have poor academic achievement, symptoms of inattention, and internalized behavioral problems [17]. Further, there is evidence to suggest that LBW is linked to a higher risk of illness, including diabetes, hypertension, hyperlipidemia, and cardiovascular diseases [6, 7, 8, 9]. Taking these findings of previous studies into account, the first possible path is educational attainment. When a society is stable and affluent, there is higher job security. Nevertheless, once society becomes unstable, socially vulnerable groups are worst affected. Those who were born with an LBW may have been hit harder in times of crisis (at the earthquake and before the recovery phase), and they may not be able to catch up during the recovery phase. The second path is through health condition. Because the supply of healthcare services weakens during the aftermath of earthquake, those with limited access to proper medical care/regular checkups experience negative impacts on their health status. Health deterioration further results in a decline of one's capability to work, leading to lower income or even more unstable jobs such as part‐time work, even after regional economies are boosted.

We have one data limitation and two caveats that need to be made aware of when interpreting our results. The limitation of our data is that the birth weight may have recall bias. This possibility cannot be eliminated in our current data set, and future research in needed with recorded birth weight. Caveats were: First, the study sample is limited to national health insurance holders. The greatest difference from the population survey is that it excludes employed full‐time workers. Our results depict a picture of the self‐employed and contingent/temporary/part‐time workers with short‐term contracts. According to the Labour Force Survey, in February 2011, in Japan, 11.4% of people were self‐employed [18], and among them, 87.0% were small enterprises and 12.7% were medium enterprises [19]. The damage of the earthquake on enterprises was unprecedented, and the total amount of damage caused by the earthquake amounted to over 1 trillion in Tohoku area. Reconstruction and economic boom also happened during our survey periods. Thus, we focused on those who were very likely to be the most severely affected by the earthquake. In addition, as presented in summary statistics, our sample had a relatively lower level of education. This characteristic should also be noted. Second, there may be a survival bias. Because our sample can only include the ones who could survive throughout their lives. Hence, we are only estimating the impact of those who survived despite their unhealthy condition at birth. If this bias exists, we yielded lower bounds of the effects of LBW.

## 5 Conclusions

We focused on the middle‐aged, national health insurance holder males living in the Miyagi and Iwate prefectures between 2013 and 2016. The massive earthquake brought about large‐scale destruction in the area, which caused great economic damage and shortage in healthcare services, followed by economic boom induced by reconstruction demand. We found that respondents with VLBW, in particular, were not being benefitted from the economic recovery. This might be attributed to the coupling of the negative impact of the earthquake with immediate economic destruction and limited access to healthcare services, which made it difficult for them to catch up. Considering the fact that our current society is undergoing both economic instability and shortage in healthcare services, owing to the COVID‐19 pandemic, this study has important implications; it is critical to support the vulnerable people during the recovery phase postcrisis.

## Author Contributions

**Midori Matsushima:** conceptualization (lead), formal analysis (lead), funding acquisition (equal), software (equal), validation (equal), visualization (equal), writing–original draft (lead), writing–review and editing (supporting). **Taku Obara:** conceptualization (supporting), data curation (equal), formal analysis (supporting), funding acquisition (lead), investigation (equal), methodology (supporting), project administration (lead), resources (lead), software (equal), supervision (lead), validation (equal), visualization (supporting), writing–original draft (supporting), writing–review and editing (lead). **Mami Ishikuro:** conceptualization (supporting), data curation (lead), formal analysis (supporting), investigation (supporting), methodology (supporting), project administration (supporting), resources (equal), software (equal), validation (supporting), visualization (supporting), writing–original draft (supporting), writing–review and editing (supporting). **Naoki Nakaya:** conceptualization (supporting), data curation (equal), formal analysis (equal), funding acquisition (lead), investigation (equal), methodology (supporting), project administration (supporting), resources (equal), software (equal), supervision (equal), validation (equal), visualization (supporting), writing–original draft (supporting), writing–review and editing (supporting). **Atsushi Hozawa:** conceptualization (equal), data curation (equal), formal analysis (equal), funding acquisition (lead), investigation (equal), methodology (supporting), project administration (supporting), resources (equal), software (supporting), supervision (equal), validation (equal), visualization (supporting), writing–original draft (supporting), writing–review and editing (supporting). **Shinichi Kuriyama:** conceptualization (supporting), data curation (equal), formal analysis (supporting), funding acquisition (lead), investigation (equal), methodology (supporting), project administration (supporting), resources (equal), software (supporting), supervision (supporting), validation (equal), visualization (supporting), writing–original draft (supporting), writing–review and editing (supporting).

## Ethics Statement

Ethics Committee of University of Tsukuba, Faculty of Humanities and Social Sciences (Reference no: 2019‐03). Ethics Committee of Tohoku University Tohoku Medical Megabank Organization (Reference no.: 2020‐4‐019).

## Conflicts of Interest

The authors declare no conflicts of interest associated with this manuscript.

## Previous Presentations

This work was presented in the 17th Annual Conference of the Japan Health Economic Association.

## Data Availability

The data that support the findings of this study are not publicly available due to their containing information that could compromise research participant consent. All inquiries about access to the data should be sent to the TMM (dist@megabank.tohoku.ac.jp).
